# Significantly altered peripheral blood cell DNA methylation profile as a result of immediate effect of metformin use in healthy individuals

**DOI:** 10.1186/s13148-018-0593-x

**Published:** 2018-12-13

**Authors:** Ilze Elbere, Ivars Silamikelis, Monta Ustinova, Ineta Kalnina, Linda Zaharenko, Raitis Peculis, Ilze Konrade, Diana Maria Ciuculete, Christina Zhukovsky, Dita Gudra, Ilze Radovica-Spalvina, Davids Fridmanis, Valdis Pirags, Helgi B. Schiöth, Janis Klovins

**Affiliations:** 1 Latvian Biomedical Research and Study Centre, Ratsupites Str. 1 k-1, Riga, LV-1067 Latvia; 2 Riga East Clinical University Hospital, 2 Hipokrata Street, Riga, LV-1038 Latvia; 3 Department of Neuroscience, Functional Pharmacology, Uppsala University, BMC, Box 593, 751 24 Uppsala, Sweden

**Keywords:** Metformin, Epigenetics, DNA methylation, White blood cells, Longitudinal study

## Abstract

**Background:**

Metformin is a widely prescribed antihyperglycemic agent that has been also associated with multiple therapeutic effects in various diseases, including several types of malignancies. There is growing evidence regarding the contribution of the epigenetic mechanisms in reaching metformin’s therapeutic goals; however, the effect of metformin on human cells in vivo is not comprehensively studied. The aim of our study was to examine metformin-induced alterations of DNA methylation profiles in white blood cells of healthy volunteers, employing a longitudinal study design.

**Results:**

Twelve healthy metformin-naïve individuals where enrolled in the study. Genome-wide DNA methylation pattern was estimated at baseline, 10 h and 7 days after the start of metformin administration. The whole-genome DNA methylation analysis in total revealed 125 differentially methylated CpGs, of which 11 CpGs and their associated genes with the most consistent changes in the DNA methylation profile were selected: *POFUT2*, *CAMKK1*, *EML3*, *KIAA1614*, *UPF1*, *MUC4*, *LOC727982*, *SIX3*, *ADAM8*, *SNORD12B*, *VPS8*, and several differentially methylated regions as novel potential epigenetic targets of metformin. The main functions of the majority of top-ranked differentially methylated loci and their representative cell signaling pathways were linked to the well-known metformin therapy targets: regulatory processes of energy homeostasis, inflammatory responses, tumorigenesis, and neurodegenerative diseases.

**Conclusions:**

Here we demonstrate for the first time the immediate effect of short-term metformin administration at therapeutic doses on epigenetic regulation in human white blood cells. These findings suggest the DNA methylation process as one of the mechanisms involved in the action of metformin, thereby revealing novel targets and directions of the molecular mechanisms underlying the various beneficial effects of metformin.

**Trial registration:**

EU Clinical Trials Register, 2016-001092-74. Registered 23 March 2017, https://www.clinicaltrialsregister.eu/ctr-search/trial/2016-001092-74/LV.

**Electronic supplementary material:**

The online version of this article (10.1186/s13148-018-0593-x) contains supplementary material, which is available to authorized users.

## Background

Metformin is the first-line drug for type 2 diabetes (T2D) therapy, used since 1950s [1]. Although there are a great number of various studies on the metformin pharmacogenomics, pharmacokinetics, and lately its interaction with the gut microbiome, the details of the molecular mechanisms of metformin action have not been fully understood.

So far, there are only a few studies within the context of metformin action and changes in one of the most commonly studied epigenetic modifications—DNA methylation. One of the targeted studies has shown that metformin treatment of pregnant rats with gestational diabetes can reduce methylation level of peroxisome proliferator-activated receptor γ coactivator-1A (PPARGC1A), therefore preventing the abnormal glycolipid metabolism in their offspring [2]. In addition, a genome-wide study of metformin effects on lymphoblastoid cell lines has revealed potential biomarkers for metformin’s anticancer response [3]. In the context of possible molecular mechanisms of how metformin induce changes in the methylation profile, a recent study has proved that, in cancer cells, metformin can exert its effects via regulation of the H19/SAHH axis [4]. This has been supported by data showing that metformin promotes global methylation by decreasing *S*-adenosylhomocysteine (SAH) intracellular levels in various cell types, including non-cancerous [5]. One of the latest studies have specifically shown metformin’s effect on lowering the methylation levels at the metformin transporter genes, resulting in higher expression levels in liver tissue [6]. Studies describing other epigenetic effects of metformin have shown its impact on various histone modifications via multiple mechanisms, mostly AMPK dependent, and effect on expression levels of numerous miRNAs through increase in DICER protein levels as well [7].

Nevertheless, there is a significant lack of information on how metformin affects global epigenetic regulation in non-cancerous cells or in cells obtained from metformin-treated humans. Therefore, our aim was to investigate the short-term effect of metformin on DNA methylation profiles in blood cells from healthy volunteers. Here we compared the changes in DNA methylation in the same subjects before and after the metformin intake.

## Results

### Characteristics of the study participants

We used Illumina Infinium 450k array to evaluate the effect of metformin on DNA methylation in 12 healthy volunteers. The characteristics of the study group are summarized in Table 1. Samples, for analysis of the methylation levels, from each participant were obtained at three time points, further marked as M0 (before starting a metformin therapy), M10h (10 h after the first metformin intake, before the second tablet), and M7d (time point after 7 days of metformin administration). M10h sample was chosen to evaluate effect of one metformin’s dose; to ensure accuracy of this measurement, all study participants were strictly instructed to take the second metformin tablet only after the M10h blood sampling.

**Table 1:** Characteristics of the study group

Characteristic	Value
Female/male, *n* (%)	7 (58.3%)/5 (41.7%)
Age, years, mean ± SD	31.4 ± 6.7
BMI, mean ± SD	25.3 ± 3.5
ALAT*, U/l, mean ± SD	25 ± 13
Creatinine*, μmol/l, mean ± SD	68 ± 8.9
Fasting plasma glucose*, mmol/l, mean ± SD	5.1 ± 0.3

*BMI* body mass index, *SD* standard deviation, *ALAT* alanine aminotransferase

*Samples for hematological, biochemical tests were collected before metformin administration

### Differentially methylated CpGs

**Fig. 1:**
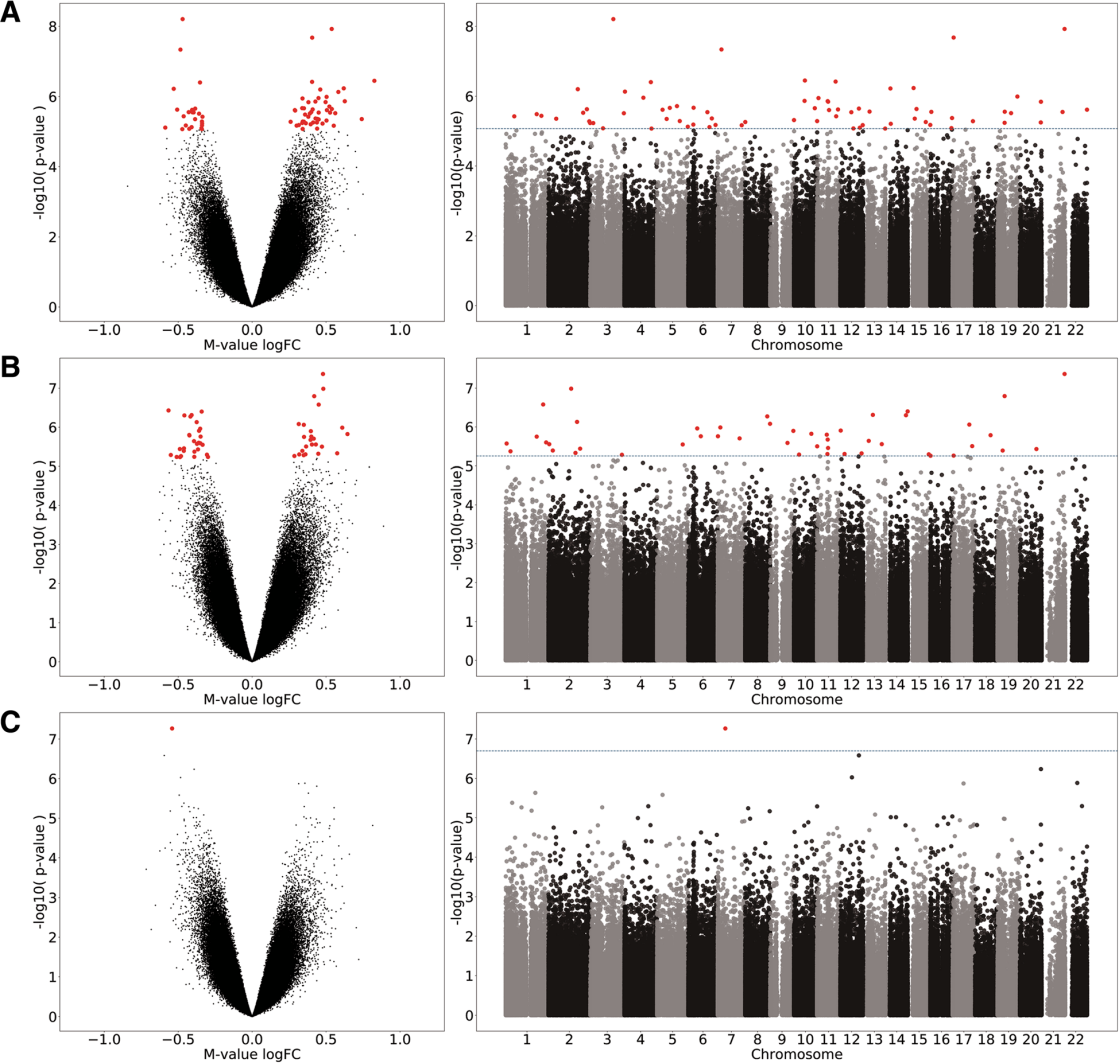
Differentially methylated positions in all analyzed contrasts. Volcano plot showing raw − log10 (*p* value) versus log-fold change of *M* values and the Manhattan plot showing the position of probes with their corresponding unadjusted *p* values across the genome in **a** M10h vs M0, **b** M7d vs M0, and **c** M7d vs M10h sample comparisons. The significant CpG sites (after FDR correction) are highlighted in red. M0—before starting a metformin therapy; M10h—10 h after the first metformin intake, before the second tablet; M7d—time point after 7 days of metformin administration

During the data preprocessing stage, 64,512 (13.29%) probes were filtered out, leaving 421,000 probes for downstream analysis. To detect differentially methylated CpG sites/probes (DMPs), we applied limma analysis between contrasts at all three time points, i.e., baseline, after 10 h and 7 days of metformin administration. The model included the methylation values at the contrasted time points, together with the cell-type estimations as covariates. Comparing methylation values at M10h and M0 samples, 72 differentially methylated CpG sites with a false discovery rate (FDR) of < 0.05 were identified after correction for multiple testing using the Benjamini-Hochberg method. In the same way, 52 DMPs were found applying contrast between methylation levels at M7d and M0 and only one (cg07026010—*NUDCD3*) in case of M7d with M10h comparison (full list of significant CpGs is available in Additional file 1). Of these, 43 (59.72%), 24 (46.15%), and 1 (100%) CpG sites were hypermethylated, and 29 (40.28%), 28 (53.85%), and 0 (0%) CpG sites were hypomethylated when contrast analyses were applied for M10h vs M0, M7d vs M0, and M7d vs M10h respectively (Fig. 1). The median absolute difference in beta values, comparing all contrasts, was 0.013 (interquartile range (IQR), 0.006–0.029) for statistically significant differentially methylated probes. The average estimated genomic inflation factor (*λ*) for all three contrasts before correction was 1.64 ± 0.28, and after including covariates, it was reduced to 1.30 ± 0.15. Additional evaluation of *λ* with qq-plots depicted the same improvement ensured by including covariates (data not shown).

**Fig. 2:**
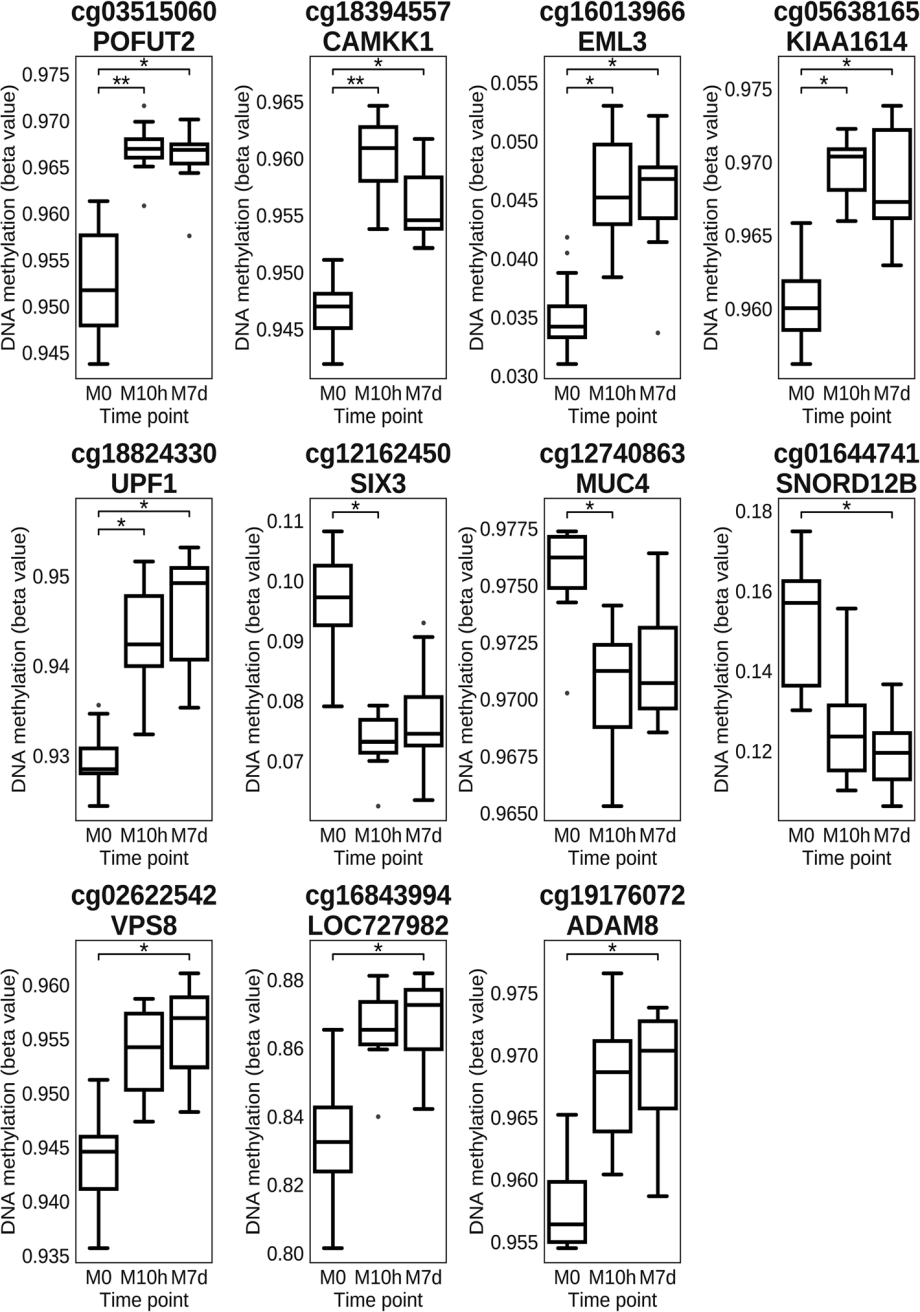
Methylation levels of the top 11 most significant CpGs across the investigated three time points, i.e., M0 (before starting a metformin therapy), M10h (10 h after the first metformin intake, before the second tablet), and M7d (time point after 7 days of metformin administration), together with their associated genes. Box plots depict median, maximum, minimum, 25th percentile, and 75th percentile. Dots beyond the bounds of the whiskers represent outliers. * and ** denote significance levels 0.05 and 0.01 respectively

Among the identified DMP, a total of 11 CpGs with the most consistent changes in the DNA methylation profile were emphasized (Fig. 2) based on two additional criteria. First, we included all overlapping DMP at both contrasts M10h vs M0 and M7d vs M0 (*n* = 5; cg03515060, cg18394557, cg16013966, cg05638165, cg18824330). Second, we selected probes if their median beta values at time points M10h and M7h overlapped IQRs of M7h and M10h, respectively. Also, IQRs of both time points could not overlap with IQR of time point M0 (*n* = 6; cg12740863, cg16843994, cg12162450, cg19176072, cg01644741, cg02622542). Of these 11 CpGs, 8 (72.73%) CpG sites displayed hypermethylation, while 3 (27.27%) CpG sites showed hypomethylation when comparing methylation levels after the metformin use (at time points M10h and M7d) with methylation levels before the use of metformin (Fig. 2).

All identified 11 CpG sites corresponded to 11 genes according to the 450k annotation file published by Price [8] (Table 2). One of these CpG sites was located in high-density CpG island, 7 CpG sites—in intermediate-density CpG islands with 1 bordering high-density CpG island, and 3—in non-islands according to HIL CpG classes.

**Table 2:** Characterization of the top 11 most significant CpG sites

Filter	CpG site	Chr	logFC M10h vs M0	logFC M7d vs M0	FDR M10h vs M0	FDR M7d vs M0	Gene	Distance to the closest TSS	Gene context^a^	Spearman’s correlation between methylation and transcription
Significant in both of the following contrasts: M10h vs M0 and M7d vs M0	cg03515060	21	0.538	0.479	*0.003*	*0.018*	POFUT2	1984	Body	− 0.184
	cg18394557	17	0.406	0.286	*0.003*	*0.047*	CAMKK1	− 8799	Body	− 0.042
	cg16013966	11	0.428	0.395	*0.034*	*0.037*	EML3	− 308	1stExon;5’UTR;TSS1500	− 0.23
	cg05638165	1	0.358	0.351	*0.035*	*0.034*	KIAA1614	14,198	Body	− 0.337
	cg18824330	19	0.363	0.419	*0.043*	*0.022*	UPF1	− 9944	Body	− 0.382
Significant in one of the contrasts and medians for time points M10h or M7d in IQR	cg12740863	3	− 0.359	− 0.260	*0.034*	0.127	MUC4	− 26,158		0.37
	cg16843994	2	0.347	0.404	0.091	*0.038*	LOC727982	− 706		NA
	cg12162450	2	− 0.386	− 0.349	*0.040*	0.059	SIX3	7515		NA
	cg19176072	10	0.462	0.472	0.054	*0.040*	ADAM8	5756	Body	− 0.312
	cg01644741	20	− 0.287	− 0.366	0.137	*0.043*	SNORD12B	39	Body,TSS1500	0.036
	cg02622542	3	0.269	0.348	0.151	*0.047*	VPS8	− 2419		0.166

Statistically significant FDR values are marked in italics

*5′UTR* 5′ untranslated region, *TSS* transcription starting site

^a^TSS1500: Region 200–1500 base pairs upstream of the transcription start site

To analyze the possible influence of circadian changes on the methylation profile, firstly, we searched our DMP list for the most common genes associated with regulation of circadian rhythm, such as *BMAL1*, *PER1*, *PER2*, *PER3*, *ARNTL*, *CRY1*, and *CRY2*. Secondly, we evaluated the main known functional roles of genes associated with the 125 DMPs, and, thirdly, we used the results from pathway enrichment analysis to find any connections with the circadian regulation. In result of these steps, we did not find any significant associations between the DMPs and circadian rhythm.

The correlation between methylation and RNA expression level of the corresponding gene was verified using targeted data form RNA-seq. Out of 11 genes tested, only the expression of *UPF1* (*p* − 0.024), *MUC4* (*p* − 0.029), and *KIAA1614* (*p* − 0.048) showed significant correlation with the methylation of corresponding CpG sites (Table 2).

### Differentially methylated regions (DMRs)

During the DMR analysis, we found 13 regions with significant differences in methylation levels (summarized in Table 3). Five of the identified regions overlapped with some of the significant DMPs but not with the 11 sites prioritized by us.

**Table 3:** Differentially methylated regions

Contrast	Gene	FDR	Number of probes	Chr	Start (bp)^a^	End (bp)^a^	Transcription factors^b^
M10h vs M0	*EPHB1*	1.60E−11	3	3	134,515,421	134,516,302	–
	*CDCA7L*	3.83E−07	5	7	21,985,276	21,985,628	Nr1h3
	*CLVS2*	8.21E−07	10	6	123,317,123	123,317,875	Nrsf
	*BACE2*, *MIR3197*	1.38E−06	3	21	42,539,960	42,540,409	CTCF
	*EXPH5*	5.76E−06	6	11	108,464,101	108,464,498	Cmyc; Egr1; FOXA1; MYC; Max; SP1;
	*KCNE4*	1.50E−05	3	2	223,916,686	223,916,861	USF1
	*TTC38*	1.50E−05	4	22	46,685,471	46,685,728	NA
	*TTC39A*	1.51E−05	5	1	51,810,626	51,811,022	–
	NA	2.17E−05	3	4	153,897,215	153,897,453	NA
	NA	2.33E−05	3	10	132,891,318	132,891,371	NA
M7d vs M0	*SFRP2*	1.18E−11	28	4	132,891,371	154,711,183	CTCF; Egr1
	*GPR19*	4.59E−10	11	12	12,848,515	12,849,588	E2F4; ZBTB33;
	*TMEM216*	3.46E−07	7	11	61,159,601	61,159,837	CTCF; Egr1; Gabp; Yy1

^a^Physical position (basepair, hg37)

^b^Data from Ensembl 91 regulation resources [98], hg38

### Enrichment analysis

To evaluate the potential biological significance of the impact of differentially methylated CpG sites, we performed a gene set pathway enrichment analysis by using the Ingenuity Pathway Analysis (IPA). All genes associated with significant differentially methylated probes (FDR < 0.05) from different contrasts were selected. Thus, 72 genes were selected from the M10h vs M0 contrast and included in the first pathway analysis, and 52 genes from the M7d vs M0 contrast and included in the second pathway analysis. We did not include the only significant result from the M7d vs M10h contrast. The top enriched canonical pathways are summarized in Table 4.

**Table 4:** Top enriched canonical pathways by IPA

Contrast	Pathway	*p* value
M10h vs M0	Unfolded protein response	8.82 × 10^−3^
	Salvage Pathways of Pyrimidine Deoxyribonucleotides	0.021
	Glycogen Degradation II	0.031
	Glycogen Degradation III	0.036
	Granzyme B Signalling	0.041
	Gα12/13 Signalling	0.046
	Lipid Antigen Presentation by CD1	0.048
M7d vs M0	*S*-Methyl-5-thio-α-d-ribose 1-phosphate Degradation	6.82 × 10^−3^
	Gustation Pathway	0.025

**Fig. 3:**
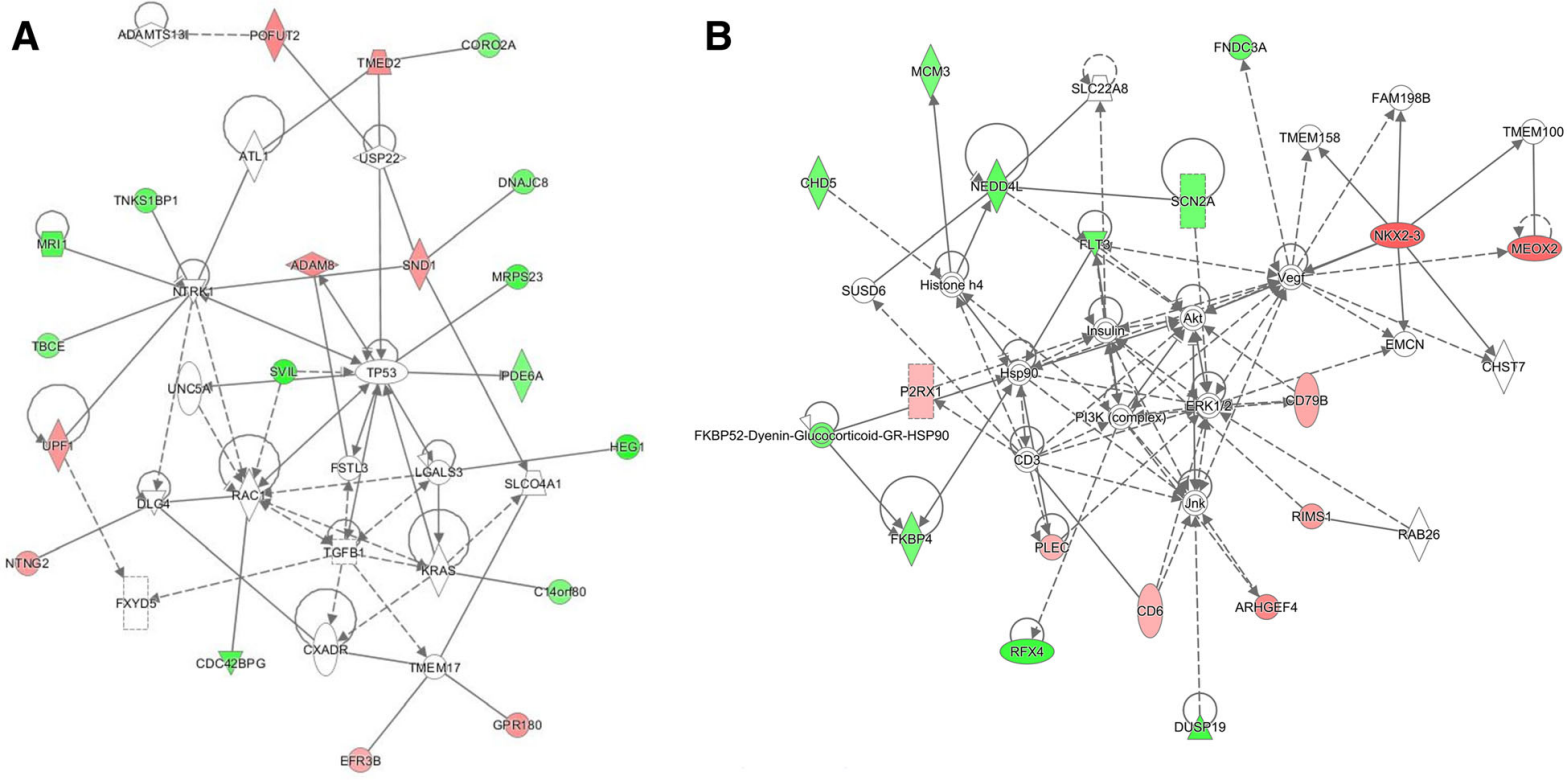
Top enriched networks from IPA. Green nodes—hypermethylated; red nodes—hypomethylated. **a** Cell-to-cell signaling and interaction, cellular assembly and organization, and cellular function and maintenance (central predicted associated biological functions—tumorigenesis processes) had an IPA score of 48. **b** Cell morphology, cell-to-cell signaling and interaction, and cellular assembly and organization (central predicted associated biological functions—metabolism processes) had an IPA score of 41

In addition to the canonical pathways, we identified nine enriched networks in the M10h vs M0 contrast, and four in the M7d vs M0 comparison. The top enriched networks with IPA score > 20 were as follows (score/focus molecules): M10h vs M0—hematological system development and function, cellular movement, cell-to-cell signaling and interaction (28/13); hereditary disorder, neurological disease, organismal injury and abnormalities (23/11). M7d vs M0—cell-to-cell signaling and interaction, cellular assembly and organization, cellular function and maintenance (48/19); cell morphology, cell-to-cell signaling and interaction, cellular assembly and organization (41/17). Two of the most relevant networks are visualized in Fig. 3.

## Discussion

The aim of our study was to examine metformin-induced alterations in epigenetic regulation processes by performing genome-wide DNA methylation analysis in human white blood cells followed by estimation of RNA expression levels of identified genes. We conducted our study in order to understand the pathways affected by metformin at real life physiological conditions in humans. This is extremely important taking into account the pleiotropic effects of metformin, and such studies may pinpoint important novel targets not only for treatment of T2D but also for other diseases. Various studies have shown that the evaluated effects in the methylation profile of peripheral blood DNA, that is the only option to access repeated tissue sampling in humans, are highly representative to the changes in other organs [9–11]. It is known that the DNA methylation pattern is highly subject specific and is influenced by many factors making it very difficult to identify the metformin-specific effects in a case-control-based type of study. We therefore selected a longitudinal design for this study, using short response time in order to exclude the influence of other factors. We also involved healthy volunteers to avoid a background of any commonly studied diseases so far related with the beneficial effects of metformin. One of our goals was to detect the fastest practically measurable effect of metformin on DNA methylation. Taking into account the known high variability of metformin pharmacokinetics, the time point when to evaluate the immediate and at the same time most profound effect was chosen to be the impact of one dose, and sampling time was selected at 10 h, before the recommended administration time of the second dose. Furthermore, M10h vs M0 sample comparison revealed the highest number or DMPs, representing the significant effect of one metformin dose.

To our best knowledge, this is the first study showing the metformin-mediated change of DNA methylation in healthy individuals already 10 h after administration. From the pool of 125 significantly modified sites, we prioritized 11 differentially methylated CpG with the largest and most consistent changes in beta values at different contrasts.

We assumed that some methylation changes measured at 10 h (M10h) could be caused by the circadian rhythm, which has been well described before and proven to be a driver of dynamic gene expression [12]. To avoid any false conclusions about the epigenetic targets of metformin, we paid specific attention to the presence of genes involved in the circadian rhythm among regions covering DMPs. We also evaluated this possibility by focusing on two contrasts that represent the different methylation profiles of DNA purified from blood samples that were collected in two distinct time points of the day—M7d vs M0 and M7d vs M10h. We did not observe any overlapping DMPs between the particular contrasts, suggesting no significant influence of the circadian rhythm on the DNA methylation in our data. Surprisingly, we observed only one significant DMP comparing M7d and M10h time points, providing a strong support for the fact that observed methylation changes are indeed caused by metformin rather than other factors changing during the trial, such as diet or circadian cycle.

Genes corresponding to the top-ranked DMPs represent the main functional groups associated with previously described targets of metformin therapy: regulatory processes of energy homeostasis, inflammatory responses, tumorigenesis, and neurodegeneration. The criteria based on the comparison of median beta values and IQRs (see the “Results” section) were chosen to avoid bias in prioritization and would allow to include potentially important DMPs in addition to only those being significant at both M10h vs M0 and M7d vs M0 contrasts.

Interestingly, we found DMP within *CAMKK1* gene—one of two highly homologous genes coding for Ca2+/calmodulin-dependent protein kinase kinases (CaMKK) [13]—with CaMKK2 being a known regulator of AMP-activated protein kinase (AMPK). Despite the fact that only CaMKK2 has been proven to form a stable complex with AMPK, both isoforms of the CaMKK are capable of phosphorylating the AMPKα subunit at Thr-172 in vitro [14, 15]. From our data, the differentially methylated CpG close to the *CAMKK1* TSS together with negatively correlated mRNA expression data as the result of metformin administration suggests a potential contribution of CaMKK1 in the AMPK-mediated mechanism of metformin anti-diabetic action.

Furthermore, it is known that metformin exerts its effects also via AMPK-independent mechanisms [16], as shown by CaMKK1 ability to mediate glucose uptake in muscle cells independently from AMPK and Akt [17], in that way suggesting that methylation level changes in CaMKK1 could be a part from an alternative pathway responsible for the therapeutic effects of metformin.

Additionally, we identified a differentially methylated CpG site near the transcription factor coding gene *SIX3* [18]. Downregulation of *SIX3* due to the methylation of the *SIX3* promoter is observed in lung adenocarcinoma tissues and lung cancer cell lines, where mRNA expression of the gene is also associated with higher survival rate [19]. Some research suggest SIX3 linkage to diabetes from genetic studies [20] and show *SIX3* as possible regulator of insulin production in β-cells in an age-dependent manner [21]. Lowered methylation level of CpG near the *SIX3* TSS shown in our data suggests the DNA methylation as another potential epigenetic mechanism involved in *SIX3* expression regulation. *SIX3* is not expressed in human white blood cells [22], explaining the absence of reads corresponding to *SIX3* in our RNA-seq data, but gene expression may manifest in other tissues. So far, normalized insulin production itself has not been considered as a therapeutic effect of metformin, although it might be affected along with metformin-induced improvements of insulin sensitivity [23].

Our data also show ADAM8 as a considerable potential contributor in the anti-inflammatory action of metformin, that is, one of the known beneficial effects of this medication [24]. ADAM8 is a cell surface protease, mainly expressed in granulocytes and monocytic cells, where it conducts the regulation of monocyte adhesion and migration [25–27]. Its contribution in the inflammatory responses regarding neurodegenerative disorders, allergy, asthma, and acute lung inflammation has been widely described before [28–31]. Our data justify the anti-inflammatory properties of metformin independently of diabetes status [24] and suggest the potential contribution of ADAM8 in the process. Due to its expression in human white blood cells, ADAM8 might be considered a promising biomarker for the detection of metformin-induced anti-inflammatory responses while reflecting inflammatory processes in adipocytes; however, further experimental evidence is required.

Many of the genes linked to the top-ranked DMPs are functionally associated with various malignancies. The most significant DMP in our study appeared to be situated in the body of *POFUT2*. O-Fucosyltransferase 2 encoded by *POFUT2* is proved to restrict epithelial-mesenchymal transition and affect cell motility in mouse embryos [32], and is considered as a useful prognostic biomarker in patients with glioblastoma and adenocarcinoma [33, 34]. To our knowledge, there are no reports yet describing *POFUT2*’s association with the beneficial effects of metformin. Our data also show several more DMPs located within or near the TSS of tumor-related genes, including SNORD12B—previously associated with colorectal and breast cancer pathogenesis [35–37], MUC4—promising prognostic marker and therapeutic target in the case of pancreatic cancer [38–40], KIAA1614 with promoter hypermethylation observed in colon tissues from patients with ulcerative collitis as well as in colon cancer cell lines [41], and UPF1 with indisputably crucial role in the maintenance of genome stability, significantly implicated in various malignancies [42–47].

The functions of two genes from the top DMPs’ associated list are poorly defined. We identified increased DNA methylation level close to the TSS of VPS8 gene. VPS8 is an accessory subunit of CORVET complex, necessary for mediating multiple steps in the endocytic pathway and required for fusion of early endosomes [48]. Thus far, there is no conclusive data indicating the possible effects of VPS8 dysregulation on phenotype in humans [49–52]. Likewise, the function of long intergenic non-protein coding RNA 1249 (LINC01249/LOC727982) is still not clear with only few reports on genetic association of SNPs within the gene with infectious disease and blood pressure [53, 54].

Overall, the DNA methylation has a repressive effect on transcription factor binding; therefore, we used ENCODE data on transcription factor binding sites to identify such possible interactions [55, 56]. We detected transcription factors CTCF, CTCFL, and Egr1 binding to the genomic region overlapping the differentially methylated CpG within *EML3* gene; out of these, CTCF is proved to mediate glucagon production [57] and Egr1 is responsible for insulin resistance [58]. Although there are no data available to date, supporting direct metformin impact on EML3 (nuclear microtubule-binding protein) [59] or describing EML3 contribution in metformin therapeutic effects, increased expression of *EML3* in cultured human cells as a result of metformin-1816 small molecule perturbation has been reported before [60]. Likewise, the genomic region within *UPF1* gene, covering the top-ranked CpG site is associated with CTCF, Egr1, and two more transcription factors: MYC—involved in the pathogenesis of diabetes [61], and PU1—initiating insulin resistance as well as regulating lipolysis [62].

The detected DMRs, as well, could essentially be grouped by connection to the processes currently known to be affected by metformin. For example, the most significant DMR was associated with *EPHB1*, which together with other Ephrin receptors forms the largest subgroup of the Eph receptor tyrosine kinase (RTK) family [63]. Underexpression of the EphB1 protein is significantly associated with tumor progression in gastric carcinomas and higher invasiveness of colorectal cancer cells, suggesting a tumor-suppressive role of the protein and possible implication in the beneficial effects of metformin [64, 65].

Another noteworthy DMR was associated with APP-cleaving enzyme 2 coding gene (*BACE2*) encoded protein that cleaves amyloid precursor protein into amyloid beta peptide, and is implicated in the pathogenesis of neurodegenerative diseases including Alzheimer’s disease [66–68]. Interestingly, increased β-cell proliferation and glucose-stimulated insulin secretion resulting from reduced Bace2 levels have been previously reported [69]. In a mouse model of T2D, induced by the overexpression of human islet amyloid polypeptide, *BACE2* deficiency improved glucose tolerance, suggesting that *BACE2* inhibition might serve as a potential therapeutic strategy for T2D treatment [70].

Another DMR is associated with *SFRP2*, Secreted Frizzled Related Protein 2. Methylation changes in the promoter region of *SFRP2* have been proposed as a potential noninvasive biomarker for colorectal cancer [71, 72]. Its mRNA is also expressed in mouse and human adipose tissue, and elevated levels have positive correlation with BMI and with abnormal glucose tolerance [73].

The pathway enrichment analysis revealed metformin’s association with various pathways some of which already has been described in connection with metformin action but not in the context of epigenetic regulation. The top enriched pathway after one dose of metformin—Unfolded Protein Response (UPR)—has been shown to be one of the main mechanisms of inducing apoptosis by metformin in acute lymphoblastic leukemia cells [74], and metformin-induced UPR inhibition in kidney cells can explain metformin’s beneficial effects [75].

One of the products of the top enriched pathway describing changes after week long metformin administration (*S*-methyl-5-thio-α-d-ribose 1-phosphate Degradation) is l-methionine, an essential amino acid in human organism. Moreover, it is known that l-methionine is used for generation of *S*-adenosylmethionine (SAM) [76], which has been depicted to be an essential part of metformin-induced increase in global methylation levels as it accumulates in cells during metformin therapy [5]. Taking into account the results from enriched pathways and the fact that we mostly observe metformin-induced hypermethylation than hypomethylation, it is possible that activation of this particular canonical pathway may contribute to the previously described increase in SAM levels.

Although enriched networks (Fig. 3) are not directly related to known metformin effects, the downstream molecules of those associated with differential methylation levels in our study group are known to be involved in various pathways related with T2D (e.g., AKT, ERK1/2, JNK, P13K), insulin regulation processes [77], cancer development mechanisms [78], and other.

The correlation between DNA methylation and gene expression is complex and nonlinear [79]. The generally accepted consequence of DNA methylation is transcriptional repression; however, methylation in the transcribed region might also demonstrate positive correlation with mRNA expression [80]. In our study, we did not detect a convincing correlation between DNA methylation of top-ranked loci and transcription level of corresponding genes; however, the influence of methylation as well as gene expression itself are tissue-specific and might be missed by focusing on single type of cells only. Nevertheless, the significant correlation observed between the expression levels of *UPF1*, *MUC4*, *KIAA1614*, and the methylation level of the corresponding CpG sites provide evidence for a crucial contribution of epigenetic regulation in the mechanism of action of metformin, which results in specific alterations of gene expression profiles.

Currently, it is not fully known whether metformin has only an indirect effect on the epigenetic regulation processes in the human organism via the previously described H19/SAHH axis or through linking cellular metabolism to the mechanisms needed for DNA methylation [4, 5]. However, the methylation profile and concentration of metformin used in cell type specific in vitro experiments may significantly differ from the physiological levels and observations in the affected cells in human body. The large variation of SAH and SAM levels in various cell types has been described [5]. In addition, the previous studies evaluating the metformin-induced methylation profile changes mostly have been targeted; thus, it is not surprising that we did not observe the DMPs at the same genes or pathways.

Major limitation of this study is the low sample size even though there are number of reports using the same number of individuals in their studies [81–84]. On the other hand, we believe that this weakness is compensated by the number of strengths in our design. First, we used a longitudinal study design and it has been recognized that, in similar time series studies, individuals can be treated as their own controls before and during treatment and sufficiently increase the power of the study [85] compared to case-control design especially accounting for the inter-individual variability among study participants. Secondly, the longitudinal design combined with observation of methylation changes in the shortest possible time allows us to minimize the effects of other factors that can induce changes in methylation unrelated to the metformin treatment. Thirdly, inclusion of healthy subjects should have minimized false associations and conclusions arising from unaccounted treatment status by metformin or other medications in T2D patients, including the unknown true duration of T2D before diagnosis. Finally, the use of genome-wide methylation analysis allows us to observe unbiased effects and find new metformin targets.

Another limitation in our study is the lack of clinical and biochemical measures at all time points. In the same time, it has been previously shown that metformin has small or no effect of such measures as plasma glucose level in healthy individuals [86, 87], and we decided not to include those in study protocol.

Unfortunately, due to the lack of similar studies, we were not able to support our findings from literature and replication in other cohorts is needed.

## Conclusions

This is the first study showing the immediate effect of metformin on white blood cell DNA methylation in humans at therapeutic doses. The gained knowledge about the metformin-induced methylation profile changes in healthy individuals can be used as basis for further in vitro and in vivo studies, which are important due to the growing number of various metformin therapeutic application possibilities in non-diabetic patients.

## Methods

### Study design

Study group involved 12 healthy metformin-naïve voluntary individuals. The involvement and sample collection was organized in collaboration with the Genome Database of Latvian Population (LGDB) [88]. Exclusion/inclusion criteria (Additional file 2) were defined according to the requirements of concurrently ongoing clinical trial (registration number: 2016-001092-74 (www.clinicaltrialsregister.eu)), which also involves gut microbiome analysis. Participants were included if they matched the following criteria: have not used antibiotics, immunosuppressive medicaments, corticosteroids, or pharmaceutical-grade probiotics during the time period of the past 2 months; have not been diagnosed with oncological, autoimmune, chronical gastrointestinal tract diseases, or T2D; have not had diarrhea in the past week; and are not taking any other medications incompatible with metformin. The research subjects received an 850-mg metformin tablet (Berlin-Chemie AG) twice a day for a week. Samples for hematological, biochemical tests were collected in certified clinical laboratory at fasting state 1–3 days before starting the metformin administration. Whole blood samples for methylation analysis were collected at three time points: (1) before starting metformin therapy (morning, fasting state)—M0, (2) 10 h after first metformin intake, before the second tablet (evening)—M10h, and (3) after 7 days of metformin administration (morning, fasting state)—M7d. Throughout the article, we have defined the measurement of 10-h sample as the immediate effect of metformin.

### Sample analysis

DNA isolation from whole blood samples using the phenol-chloroform extraction method was performed by Genome Database of Latvian Population (briefly described before [89]). DNA samples were quantified with Qubit® 2.0 Fluorometer using Qubit dsDNA HS Assay Kit (TherfmoFisher Scientific, USA). For the bisulfite conversion, the EZ DNA Methylation-Gold TM kit (Zymo research, USA) was used according to the manufacturer’s instructions. DNA methylation was determined by the Illumina Infinium HumanMethylation450 BeadChip Array (Illumina, USA), using 500 ng of each bisulfite-treated DNA sample.

Total RNA was isolated from whole blood samples using PerfectPure RNA Blood Kit (5Prime GmbH, Hamburg, Germany). Ribosomal RNS depletion was done with Low Input RiboMinus™ Eukaryote System v2 (Thermo Fisher Scientific, USA). For cDNA library preparation, we used Ion Total RNA-Seq Kit v2 (Thermo Fisher Scientific, USA), and sequencing was performed on the Ion Proton™ System and Ion PI™ Chip (Thermo Fisher Scientific, USA).

### Data preprocessing and statistical analysis

IDAT files were imported using R package minfi [90]. Cell counts were estimated from methylation data using Houseman algorithm [91] implemented in minfi.

Data preprocessing and normalization was done using Enmix [92]. Briefly, probes with detection *p* value > 0.05 and probes with a multimodal distribution were filtered out. Background correction was performed with the function preprocessENmix using unused color channels as a background parameter estimate. Probe intensities were normalized using a quantile normalization method, and probe type bias was adjusted using the Regression on Correlated Probes (RCP) method [93]. Probes having a SNP or single base extension annotation in CpG site were excluded. Due to interrupted use of metformin by one of the study subjects, the sample taken after 1 week of metformin administration for that particular subject was discarded.

Batch effect was removed from data using slide and subsequently subjects as covariates as they showed the strongest influence on the probe methylation variability. Batch effect was removed using ComBat [94] wrapped in the Enmix package. Differentially methylated probes between time points were identified using limma [95] on ComBat preprocessed data, adjusting for the following cell types estimated by minfi: CD8T, CD4T, NK, and Gran. Inflation factor of p-value distribution was estimated using R package GenABEL [96]. All analyses were performed using R (3.3.3).

Statistically significant DMRs were identified with DMRcate software [97], FDR < 0.05. Threshold for minimum number of probes within the region was set to three. DMRs were estimated from methylation *M* values using the individual CpG site significance threshold at FDR < 0.05. The interval between individual significant CpG sites had to be less than 1000 bp in the regions. The bandwidth scaling factor was set as suggested in the manual (*C* = 2). Regulatory information from Ensembl 91 regulation resources was added to identified DMPs and DMRs using Ensembl Regulation API [98].

Pathway enrichment analysis was performed with the IPA tool [99]. Information about enriched canonical pathways and networks was obtained performing the core analysis on all significant DMPs with FDR < 0.05.

### RNA-seq data analysis

Reads were mapped against human reference genome GRCh38, and read quantification was performed using STAR (2.5.3a) [100]. Obtained per-gene read counts were normalized using trimmed mean normalization (TMM), and counts per million (CPM) values were calculated with edgeR [101]. ComBat [94] implemented in R package sva [102] was used to adjust CPM values for subject-specific effects, and the Spearman correlation was estimated for the adjusted CPM values and the beta values for 11 selected CpG sites with SciPy [103].

## Additional files

Additional file 1: Full results representing all CpGs within the analyzed contrasts with significantly changed methylation levels, identified after correction for multiple testing using the Benjamini-Hochberg method. (XLSX 21 kb)

Additional file 2: List of inclusion/exclusion criteria. (DOCX 14 kb)

## Abbreviations

CPM: Counts per million
DMP: Differentially methylated CpG site/probe
DMR: Differentially methylated region
IPA: Ingenuity Pathway Analysis
IQR: Interquartile range
RCP: Regression on Correlated Probes
T2D: Type 2 diabetes
TMM: Trimmed mean normalization
TSS: Transcription starting site
